# Supplementary material for: Identification of photoperiod-induced specific miRNAs in the adrenal glands of Sunite sheep (Ovis aries)
Source: Front Vet Sci. 2022 Jul 22;9:888207. doi: 10.3389/fvets.2022.888207 (PMC9354845; doi:10.3389/fvets.2022.888207)
Supplement: Supplementary file 1 [file Data_Sheet_1.ZIP › Supplementary file/miRDeep2 file.zip!/miRDeep2 file/Know_Structure/SP42A1/oar-mir-493.pdf]

miRBase precursor : oar-mir-493
Total read count : 8008
oar-miR-493-5p read count : 6054
oar-miR-493-3p read count : 1954
remaining reads : 0

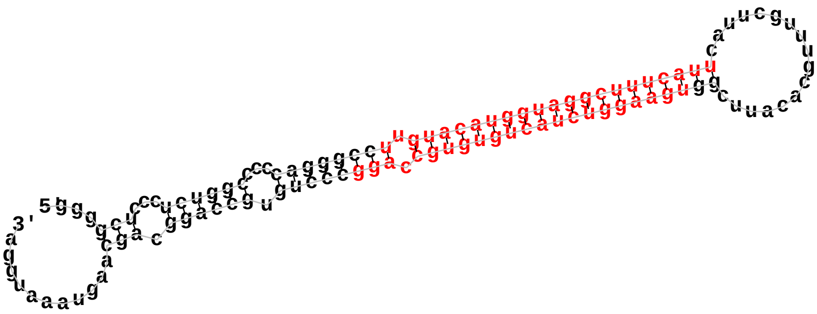

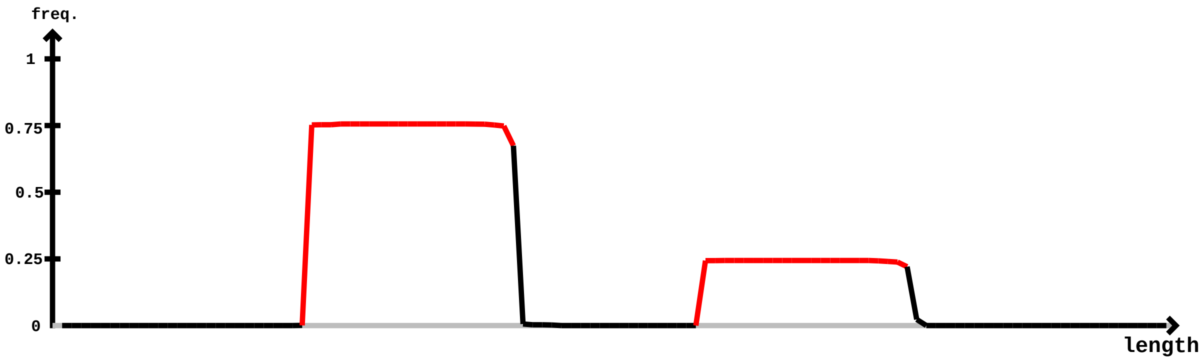

oar-miR-493-5p

oar-miR-493-3p

```
5'- ggggcucccucuggcccccagggccuuguacaugguaggcuuucauucauucguuugcacauucggugaaggucuacugugugccaggcccugugccaggcagcaaguaaaugga -3'   exp
    ...(((...((((((...((((((((.((((((((((((((((((((.................))))))))))))))))))))).)))))))).)))))).)))...........   reads   mm   sample
    ........................cuuguacaugguaggcuuucau.....................................................................   1       0    seq
    ........................cuuguacaugguaggcuuucauu....................................................................   5       0    seq
    .........................uuguacaugguaggcuu.........................................................................   4       0    seq
    .........................uuguacaugguaggcuuu........................................................................   3       0    seq
    .........................uuguacaugguaggcuuuc.......................................................................   24      0    seq
    .........................uuguacaugguaggcuuuca......................................................................   31      0    seq
    .........................uuguacaugguaggcuuucau.....................................................................   591     0    seq
    .........................uuguacaugguaggcuuucauu....................................................................   5345    0    seq
    .........................uuguacaugguaggcuuucauuc...................................................................   21      0    seq
    ..........................uguacaugguaggcuuucauu....................................................................   3       0    seq
    ..........................uguacaugguaggcuuucauuc...................................................................   2       0    seq
    ..........................uguacaugguaggcuuucauuca..................................................................   1       0    seq
    ............................uacaugguaggcuuucauucau.................................................................   5       0    seq
    ............................uacaugguaggcuuucauucauu................................................................   17      0    seq
    .............................acaugguaggcuuucauucau.................................................................   1       0    seq
    ..................................................................ugaaggucuacugugugc...............................   5       0    seq
    ..................................................................ugaaggucuacugugugU...............................   5       1    seq
    ..................................................................ugaaggucuacugugugG...............................   1       1    seq
    ..................................................................ugaaggucuacugugugcc..............................   18      0    seq
    ..................................................................ugaaggucuacugugugcca.............................   17      0    seq
    ..................................................................ugaaggucuacuAugugcca.............................   1       1    seq
    ..................................................................ugaaggucuacugugugccag............................   138     0    seq
    ..................................................................ugaaggucuGcugugugccag............................   2       1    seq
    ..................................................................ugaagAucuacugugugccagg...........................   2       1    seq
    ..................................................................ugaaggCcuacugugugccagg...........................   11      1    seq
    ..................................................................ugaaggucuacugugugccagg...........................   1553    0    seq
    ..................................................................ugaaUgucuacugugugccagg...........................   4       1    seq
    ..................................................................ugaaggucuacuguguUccagg...........................   1       1    seq
    ..................................................................ugaagguUuacugugugccagg...........................   1       1    seq
    ..................................................................ugaaggucuGcugugugccagg...........................   3       1    seq
    ..................................................................ugaaggucuacuAugugccagg...........................   4       1    seq
    ..................................................................ugaaggucuacugugugccaggc..........................   182     0    seq
    ..................................................................ugaaggucuacugugugccaggcc.........................   1       0    seq
    ...................................................................gaaggucuacugugugccagg...........................   1       0    seq
```

```
                         oar-miR-493-5p
                                                                     oar-miR-493-3p
ggggcucccucuggcccccagggccuuguacaugguaggcuuucauucauucguuugcacauucggugaaggucuacugugugccaggcccugugccaggcagcaaguaaaugga

....................................................................aaggucuacugugugccagg...........................    3          0          seq
....................................................................aaggucuacugugugccaggc..........................    1          0          seq
```
